# Atypical cutaneous manifestations of inflammatory bowel disease: A case series

**DOI:** 10.1016/j.jdcr.2026.04.065

**Published:** 2026-05-08

**Authors:** Ghida El Banna, Ramneek Kaur Dhami, Nicole Fett

**Affiliations:** a Department of Dermatology, Oregon Health and Science University, Portland, Oregon; b Department of Internal Medicine, Southern Hills Hospital and Medical Center, Las Vegas, Nevada

**Keywords:** atypical cutaneous presentations, Crohn’s disease, granulomatous lobular panniculitis, inflammatory bowel disease, nodular vasculitis, thrombotic cutaneous gangrene, ulcerative colitis

## Introduction

Patients with inflammatory bowel disease (IBD), including Crohn’s disease (CD) and ulcerative colitis (UC) often develop extraintestinal manifestations (EIMs) (reported frequency of 6% to 47%) with the most common reports of cutaneous disease.^1^ Pyoderma gangrenosum (prevalence of 0.5% to 2.6%) and erythema nodosum (prevalence of 2% to 15%) are the most common skin manifestations of IBD.^1^ These cutaneous EIMs may be concordant with or independent of the IBD activity. Rarer cutaneous EIMs also exist and can pose significant diagnostic challenges.^2^ Recognition of these rare EIMs of IBD is essential for timely diagnosis and treatment. In this case series, we describe 3 rare cutaneous manifestations of IBD seen at the Oregon Health & Science University in our dermatology-rheumatology clinic. These cases represent distinct mechanisms of IBD-associated cutaneous disease—thrombotic, granulomatous, and vasculitic—illustrating how systemic immune dysregulation in IBD can have a wide range of cutaneous manifestations. A summary of these cases is presented in Table I.

**Table I:** Summary of 3 cases of atypical cutaneous manifestations of inflammatory bowel disease

Case	IBD type	Cutaneous diagnosis	Histopathology	Treatment escalation	Outcome
23-y-old male	ulcerative colitis	thrombotic cutaneous gangrene	vascular congestion and intravascular fibrin	corticosteroids vedolizumab risankizumab	No further ischemic events
44-y-old female	Crohn’s disease	Metastatic Crohn’s	granulomatous lobular panniculitis with fat necrosis	upadacitinib	Resolution
28-y-old female	ulcerative colitis	Nodular vasculitis	superficial and deep mixed dermatitis with lobular panniculitis and ulceration	colchicine and upadacitinib	Resolution

*IBD*, Inflammatory bowel disease.

## Case 1

A 23-year-old male with psoriatic arthritis, axial spondyloarthritis, and severe, uncontrolled UC for 2 years on methotrexate and ustekinumab presented with 2 episodes of fingertip ischemia, resulting in gangrene of the left third digit. He denied any history of Raynaud’s phenomenon or triphasic color changes. On examination, all fingertips appeared dusky, and the left third fingertip showed eschar. Nailfold capillaroscopy was unremarkable. During his hospitalization for the ischemic digits, he was found to have a severe UC flare. A flexible sigmoidoscopy showed diffuse severe inflammation in the rectum and the recto-sigmoid colon. A biopsy of the rectosigmoid colon was consistent with diffuse active chronic colitis, severe, with neutrophilic cryptitis and ulcer; negative cytomegaly virus, and negative dysplasia. Patient also experienced severe anemia with hemoglobin 5.6-8.9 g/dL, ferritin 5.9, and a positive guaiac positive stool. Computed tomography of the abdomen and pelvis showed pancolitis. ESR and CRP were 24 and 33, respectively. A biopsy of the hand did not show any evidence of vasculitis and was read as vascular congestion and intravascular fibrin.

A comprehensive hypercoagulability workup, including proteins C and S, Factor V Leiden, antithrombin III (SERPINC1) gene mutation, cryoglobulins, serum protein electrophoresis, antiphospholipid antibodies, and anticardiolipin antibodies, was within normal limits. Although he tested positive for cytoplasmic antineutrophil cytoplasmic antibodies and proteinase 3 (PR3), there was no clinical or histologic evidence of systemic vasculitis; antineutrophil cytoplasmic antibodies positivity was attributed to UC.

**Fig 1:**
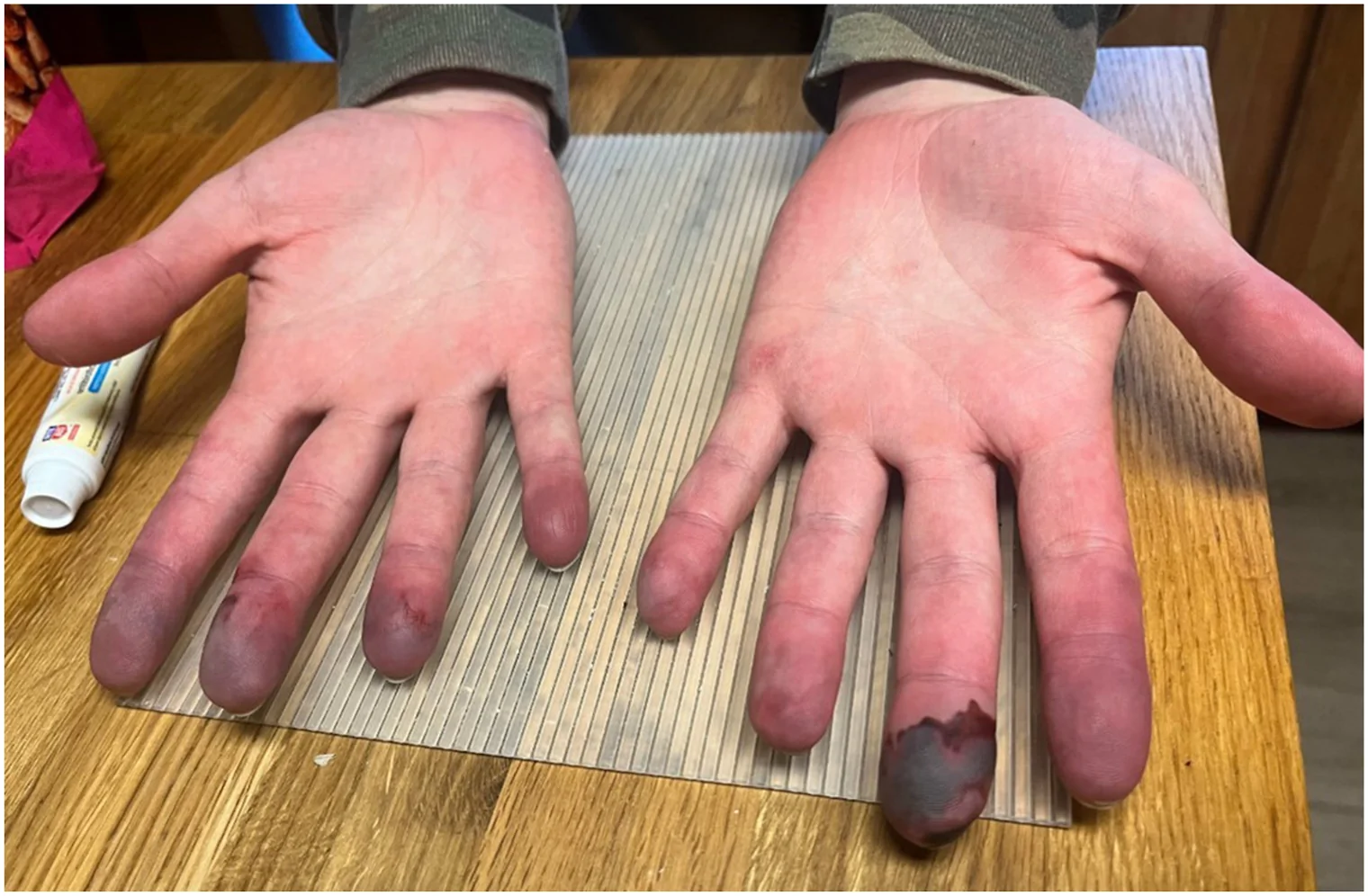
Thrombotic cutaneous gangrene of the bilateral second through fifth digits of the hands.

The clinical picture was most consistent with thrombotic cutaneous gangrene as an extraintestinal manifestation of IBD (Fig 1). His digital perfusion improved with systemic corticosteroids, and he has had no further ischemic events since transitioning to dual therapy with vedolizumab and risankizumab. Given the refractory nature of his disease, treatment with 2 biologic agents targeting distinct inflammatory pathways was medically recommended by his gastroenterology and rheumatology providers. Risankizumab was indicated to address the interleukin-23 mediated inflammatory component of his UC, psoriatic arthritis and axial disease, while vedolizumab provided complementary, gut-selective immunomodulation that was critical in controlling his systemic disease.

## Case 2

**Fig 2:**
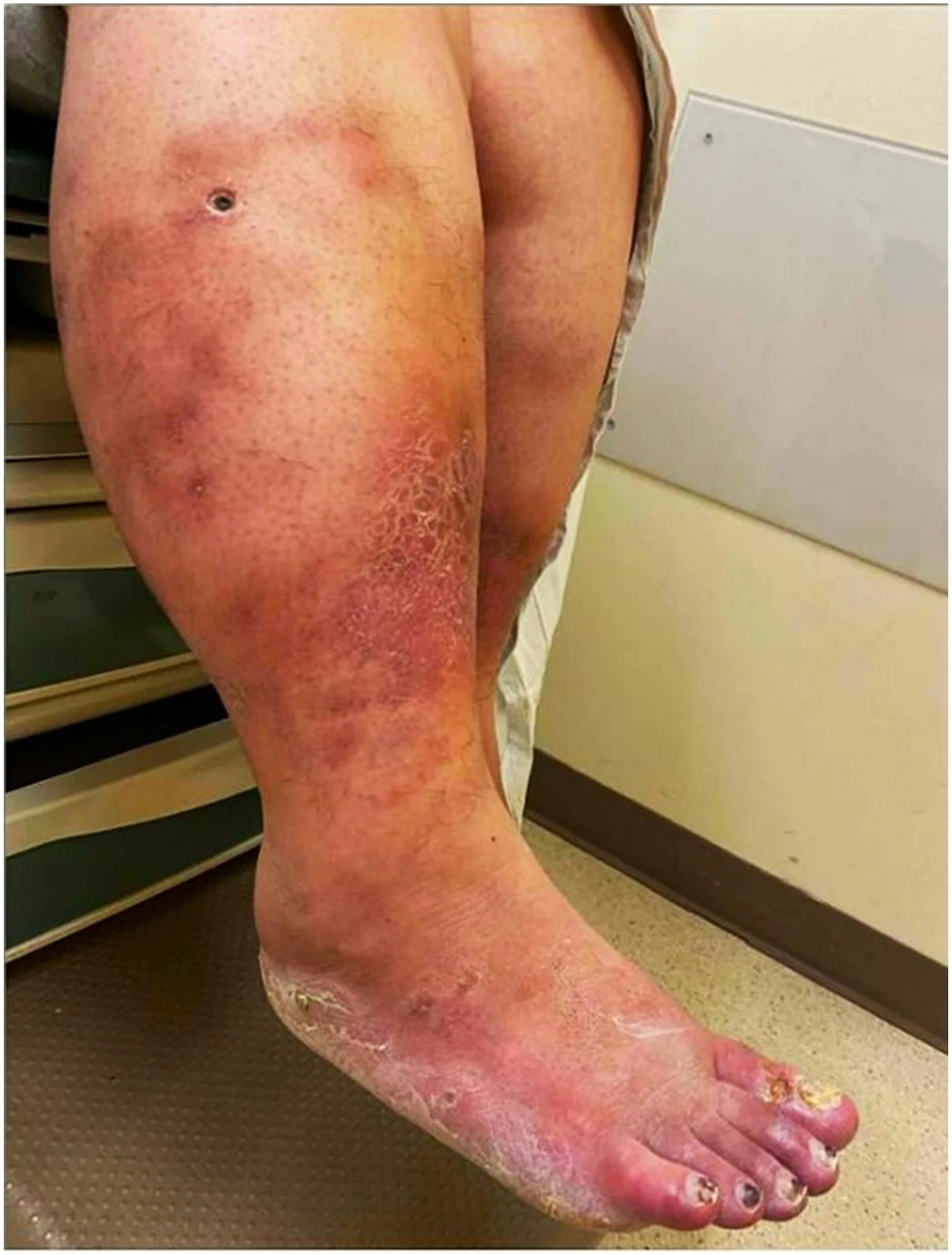
Dusky discoloration of both feet, an ulcer on the right great toe, indurated red-violaceous plaques and nodules on the lower legs, and a 5 mm shallow ulcer on the right shin.

A 44-year-old woman with rheumatoid arthritis, insulin-dependent diabetes, and severe CD for 13 years treated with ustekinumab and methotrexate. She presented with a 2-year history of tender, eroded subcutaneous nodules on the lower legs, previously treated with intermittent prednisone tapers. Patient’s colovaginal fistula symptoms remained severe despite treatment with subsequent obstruction and complications of ileostomy. At that time, a flexible sigmoidoscopy showed friability and erosions in the rectum. Upper GI endoscopy revealed no oral or esophageal ulcerations. Her CRP and ESR were elevated to 85.8 and >120, respectively. Examination revealed dusky discoloration of both feet, an ulcer on the right great toe, indurated red-violaceous plaques and nodules on the lower legs, and a 5 mm shallow ulcer on the right shin (Fig 2).

Skin biopsy demonstrated granulomatous lobular panniculitis with fat necrosis, findings consistent with a metastatic granulomatous presentation of CD. Upadacitinib 45 mg daily was added to her regimen, resulting in the resolution of the violaceous plaques and nodules within 3 months and improved control of her CD. She has remained stable on this regimen, with good control of her CD for the past 1.5 years.

## Case 3

A 28-year-old woman with hidradenitis suppurativa and untreated UC presented with a 2-year history of painful, pruritic lesions on the lower extremities. Patient had been in UC remission on infliximab for the past 8-9 years when it was stopped. About 5 months later, she started experiencing bloody stools. CRP and ESR were elevated to 86.2 and 51, respectively. Fecal calprotectin was also high at 511. Examination revealed 2-3 mm pink to violaceous papules, subcutaneous nodules, and plaques with areas of ulceration on the lower legs and feet.

**Fig 3:**
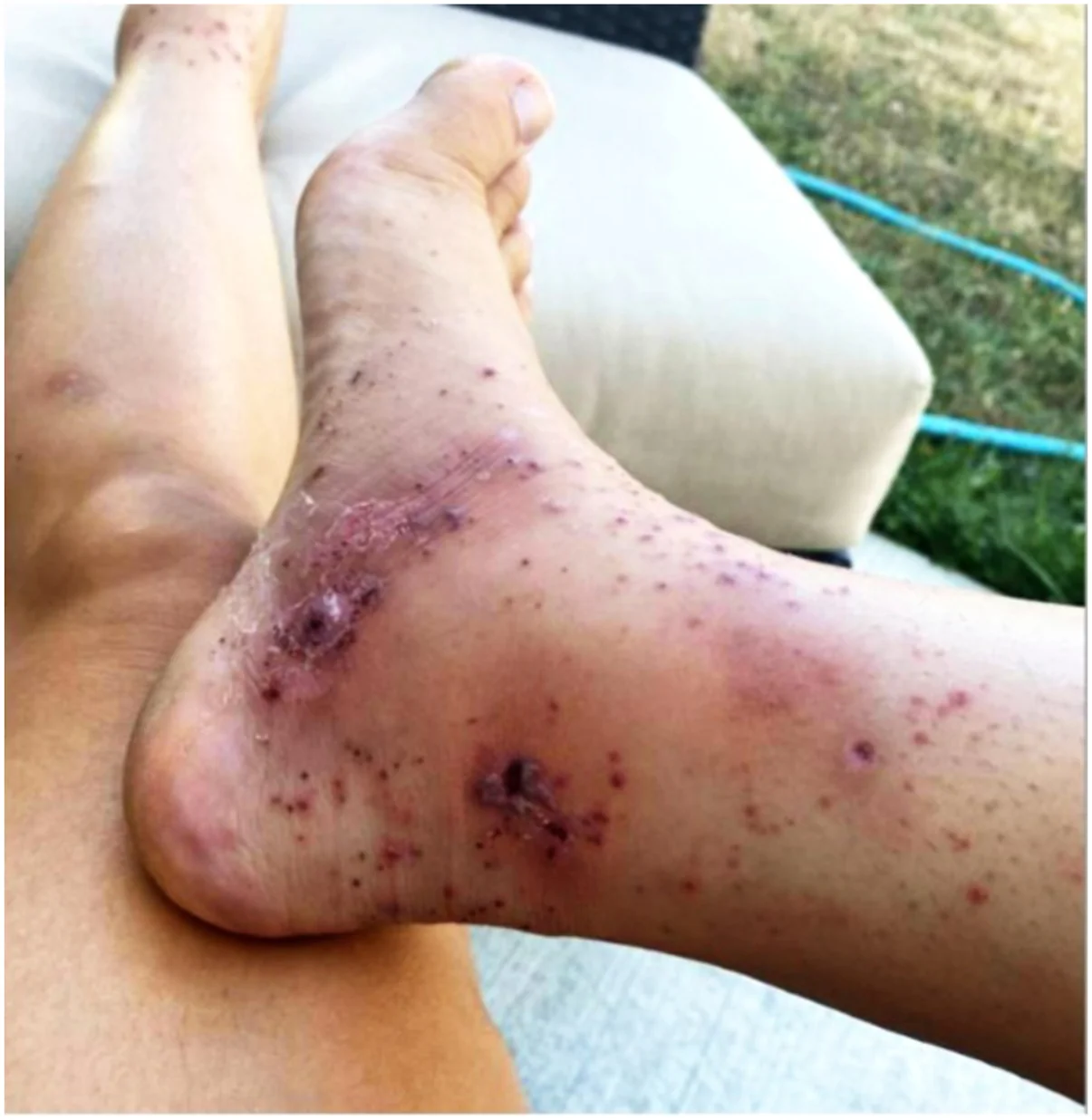
Pink to violaceous papules, nodules, and plaques with ulceration of the lower legs and feet.

Skin biopsy demonstrated superficial and deep mixed dermatitis with lobular panniculitis and ulceration. There was a dense inflammatory infiltrate composed of eosinophils and neutrophils, along with vasculitis of the small and medium vessels. Based on her clinical presentation, histologic findings, negative QuantiFERON-TB Gold, and underlying UC, a diagnosis of nodular vasculitis (NV) was made (Fig 3).

She was started on colchicine 0.6 mg daily for 1 week, increased to 0.6 mg twice daily, along with upadacitinib 45 mg daily. Her lesions resolved over 6 months, and she has remained in remission on upadacitinib 30 mg daily as a maintenance therapy.

## Discussion

These cases illustrate 3 distinct and rare cutaneous manifestations of IBD: thrombotic cutaneous gangrene in UC, granulomatous panniculitis in CD, and nodular vasculitis in UC. Each presentation highlights unique pathophysiological mechanisms through which IBD can affect the skin and underscores the importance of considering IBD-associated cutaneous manifestations in the differential diagnosis, even when presentations are atypical.

Thrombotic cutaneous gangrene, though rare, is a documented complication of UC. The hypercoagulable state associated with UC arises from multiple factors, including elevated levels of coagulation factors (Va, VIIa, VIIIa, XIa, Xa), fibrinogen, von Willebrand factor, platelets, and cryoglobulins, along with deficiencies in natural anticoagulants such as antithrombin III, protein C, and protein S.^3^ Additional contributing mechanisms include endotoxin-induced coagulation, monocyte-derived tissue factor release, and elevated homocysteine levels. During active UC flares, this hypercoagulability is believed to be driven primarily by increased platelet and von Willebrand factor levels.^3^ The elevation of von Willebrand factor may result from either direct vascular injury due to bowel inflammation or as part of an acute-phase response, in which endothelial cells are activated by inflammatory mediators.^1^^,^^2^ Treatment of thrombotic cutaneous gangrene typically includes systemic corticosteroids, anticoagulation (eg, heparin, warfarin), and escalation of IBD therapy.^1^^,^^2^^,^^4^

Panniculitides are classified histologically by the predominant location of inflammation: septal, lobular, or mixed. In IBD, the most observed form is erythema nodosum, which presents as predominantly septal panniculitis that does not ulcerate.^5^ A recent consensus statement on metastatic Crohn’s disease (MCD) defined it as the presence of genital, oral, other cutaneous disease that is not contiguous with the gastrointestinal tract.^6^, ^7^, ^8^ While this Delphi panel deemed a skin biopsy not necessary for a diagnosis of MCD, experts agreed that biopsy findings suggestive of MCD could be included as part of the minor criteria.^6^, ^7^, ^8^ These include noncaseating granulomas with any of the following: ulceration, lymphocytes and eosinophils, or vasculitis, loose granulomatous inflammation with lymphoplasmacytic inflammation, and intravascular histiocytic aggregates and perivascular or perilymphatic lymphohistiocytic granulomatous inflammation.^8^^,^^9^ In some cases, granulomatous vasculitis of small-to-medium-sized vessels can be present.^7^ The second case represents a rare presentation of MCD with ulcerating, lobular granulomatous panniculitis without vasculitis, in the setting of poorly controlled CD. The skin manifestation was not attributed to RA because patient was primarily symptomatic from her CD (abdominal pain, diarrhea, severe ileostomy symptoms) while her RA was well-controlled on ustekinumab and methotrexate. Moreover, while RA can have granulomatous cutaneous findings such as rheumatoid nodules, palisaded neutrophilic granulomatous dermatitis, interstitial granulomatous dermatitis, and granuloma annulare, they are often associated with disease severity and have distinct histopathologic findings from that of our patient. Notably, panniculitis is seen exclusively in CD and has not been reported in UC.^10^ Treatment focuses on the escalation of CD therapy.

NV is a form of lobular or mixed panniculitis with evidence of vasculitis involving small- and/or medium-sized vessels, and it is most observed in association with autoimmune conditions. NV typically presents with erythematous nodules or plaques on the posterior lower legs of young to middle-aged women.^11^ This manifestation is exceedingly rare in IBD, with only 3 cases described in the literature.^9^^,^^12^^,^^13^ All reported patients were female, aged 54 to 68 years, had CD, and were treated with monoclonal antibodies, including adalimumab and vedolizumab. At the 6-month follow-up, 1 patient achieved complete resolution; another had an unclear outcome; and the third showed no recurrence. Unlike previously reported cases of IBD-associated nodular vasculitis, which occurred in older patients with CD receiving biologic therapy, our patient was younger, had ulcerative colitis, and was untreated at presentation but experienced complete remission of NV following escalation of UC-directed therapy. This expands the clinical spectrum of NV in IBD and suggests that NV may occur in the absence of prior biologic treatment.

Although clinically heterogeneous, the cutaneous manifestations observed in this series share a common immunologic substrate linked to IBD-associated systemic inflammation. Thrombotic cutaneous gangrene reflects endothelial activation and hypercoagulability driven by inflammatory cytokines and platelet dysfunction; MCD represents a granulomatous immune response likely mediated by circulating immune complexes; and NV reflects immune-complex–mediated vascular injury within the subcutis. These patterns underscore that IBD-related skin disease may arise from vascular, granulomatous, or neutrophilic pathways.

Notably, our cases showed a positive temporal relationship between cutaneous disease and intestinal activity although the skin disease sometimes manifested years after initial diagnosis of IBD. In case 1, thrombotic ischemia occurred in the setting of uncontrolled UC and improved with escalation of IBD therapy. In contrast, case 2 demonstrated chronic cutaneous involvements paralleling refractory CD, while case 3 presented with a UC flare after stopping UC treatment following years of remission. It is important to emphasize that cutaneous manifestations do not always correlate with IBD intestinal activity as prior reports showed that they may correlate with, precede, or occur independently of intestinal inflammation, complicating diagnosis and delaying treatment.^1^^,^^9^

Importantly, all 3 patients experienced improvement or resolution of cutaneous disease following escalation of IBD-directed therapy, including biologics and Janus kinase inhibition. Upadacitinib was associated with complete remission of granulomatous panniculitis and nodular vasculitis in cases 2 and 3, respectively, emphasizing the role of Janus kinase inhibition in refractory IBD-associated cutaneous disease.

In summary, we present 3 cases to highlight the broad spectrum of cutaneous manifestations in IBD and emphasize (1) the importance of maintaining a high index of suspicion for IBD-related skin findings; (2) the diagnostic value of histopathology; and (3) the central role of treating the underlying IBD to achieve better control of cutaneous EIMs. Recognizing these associations can facilitate earlier diagnosis and appropriate treatment, ultimately improving outcomes for these challenging and often under-recognized presentations.

## Conflicts of interest

None disclosed.

## References

[1] He R., Zhao S., Cui M. (2023). Cutaneous manifestations of inflammatory bowel disease: basic characteristics, therapy, and potential pathophysiological associations. Front Immunol.

[2] Komatsu Y.C., Capareli G.C., Boin M.F.F.D.C., Lellis R., Freitas T.H.P.D., Simone K. (2014). Skin gangrene as an extraintestinal manifestation of inflammatory bowel disease. An Bras Dermatol.

[3] Singh S., Noshirwani K. (1996). An unusual cutaneous manifestation of ulcerative colitis: thrombotic skin gangrene. Postgrad Med J.

[4] Jain S., Bhatt P., Muralikrishna G.K., Malhotra P., Kumari S., Varma S. (2005). Extensive arterial and venous thrombosis in a patient with ulcerative colitis--a case report. MedGenMed.

[5] Yosipovitch G., Hodak E., Feinmesser M., David M. (2000). Acute Crohn's colitis with lobular panniculitis – metastatic Crohn's?. J Eur Acad Dermatol Venereol.

[6] Schneider S.L., Foster K., Patel D., Shwayder T. (2018). Cutaneous manifestations of metastatic Crohn's disease. Pediatr Dermatol.

[7] Park H.C., Kim H.W., Park C.G., Ko J.Y. (2016). Metastatic Crohn disease clinically reminiscent of erythema nodosum on the right leg. Cutis.

[8] Ebriani J., Santiago-Soltero K., Anshelevich E.E. (2025). Delphi panel for the development of diagnostic criteria for metastatic cutaneous Crohn disease: a consensus statement. JAMA Dermatol.

[9] Cai Z.P., Liu X., Liu W. (2023). Erythema induratum in a patient with recurrent oral ulceration: crohn's disease of the esophagus and colon. Am J Med Sci.

[10] Ko J.S., Uberti G., Napekoski K., Patil D.T., Billings S.D. (2016). Cutaneous manifestations in inflammatory bowel disease: a single institutional study of non-neoplastic biopsies over 13 years. J Cutan Pathol.

[11] Bolognia J., Cerroni L., Schaffer J.V. (2018).

[12] Misago N., Narisawa Y. (2012). Erythema induratum (Nodular vasculitis) associated with Crohn's disease: a rare type of metastatic Crohn's disease. Am J Dermatopathol.

[13] Pouldar D., Elsensohn A., Ortenzio F., Shiu J., McLeod M., De Feraudy S. (2018). Nodular vasculitis in a patient with Crohn's disease on vedolizumab. Am J Dermatopathol.

